# Profiling a Community-Specific Function Landscape for Bacterial Peptides Through Protein-Level Meta-Assembly and Machine Learning

**DOI:** 10.3389/fgene.2022.935351

**Published:** 2022-07-22

**Authors:** Mitra Vajjala, Brady Johnson, Lauren Kasparek, Michael Leuze, Qiuming Yao

**Affiliations:** ^1^ School of Computing, University of Nebraska-Lincoln, Lincoln, NE, United States; ^2^ Nashville Biosciences, Nashville, TN, United States

**Keywords:** bacterial peptide, machine learning, metagenomics, protein annotation, protein clustering

## Abstract

Small proteins, encoded by small open reading frames, are only beginning to emerge with the current advancement of omics technology and bioinformatics. There is increasing evidence that small proteins play roles in diverse critical biological functions, such as adjusting cellular metabolism, regulating other protein activities, controlling cell cycles, and affecting disease physiology. In prokaryotes such as bacteria, the small proteins are largely unexplored for their sequence space and functional groups. For most bacterial species from a natural community, the sample cannot be easily isolated or cultured, and the bacterial peptides must be better characterized in a metagenomic manner. The bacterial peptides identified from metagenomic samples can not only enrich the pool of small proteins but can also reveal the community-specific microbe ecology information from a small protein perspective. In this study, metaBP (Bacterial Peptides for metagenomic sample) has been developed as a comprehensive toolkit to explore the small protein universe from metagenomic samples. It takes raw sequencing reads as input, performs protein-level meta-assembly, and computes bacterial peptide homolog groups with sample-specific mutations. The metaBP also integrates general protein annotation tools as well as our small protein-specific machine learning module metaBP-ML to construct a full landscape for bacterial peptides. The metaBP-ML shows advantages for discovering functions of bacterial peptides in a microbial community and increases the yields of annotations by up to five folds. The metaBP toolkit demonstrates its novelty in adopting the protein-level assembly to discover small proteins, integrating protein-clustering tool in a new and flexible environment of RBiotools, and presenting the first-time small protein landscape by metaBP-ML. Taken together, metaBP (and metaBP-ML) can profile functional bacterial peptides from metagenomic samples with potential diverse mutations, in order to depict a unique landscape of small proteins from a microbial community.

## 1 Introduction

Small proteins or peptides, translated from short open reading frames, largely exist in biological systems in both eukaryotes (Chen et al., 2020) and prokaryotes (Hemm et al., 2020; Orr et al., 2021). Historically, these small proteins were ignored or identified as non-coding elements (Storz et al., 2014) and were considered as “dark matter” due to the lack of genomic annotation (Garai and Blanc-Potard, 2020). Bacteria-derived small proteins can play diverse roles in microbial functions and host-microbe interactions, such as innate immunity (Huan et al., 2020), cell division, signal transduction, transporter regulation, enzymatic activity, and protein folding (Storz et al., 2014). Some of the bacterial peptides have the potential of being novel therapeutic candidates (Duval and Cossart, 2017).

Bacterial peptides are much harder to decompose and they annotate in a natural community. While detecting and testing a small gene can be difficult in a single organism, microbiome at community level brings additional challenges in the data complexity and sparsity for small protein detection, classification, and function annotation. Metagenomics from short gun sequencing provides information from the community-specific population to gene functions, but there haven’t been many previous efforts specifically focusing on the role of bacterial peptides from a natural community. The lack of detection power and poor analytical resolution indicate the limitation from both the computation and experiment. First, the peptides detection from mass spectrometry needs abundant input materials and suffers from large search spaces in an unbiased and untargeted scenario. It usually requires a confident database from reference genomes or from metagenomes. Poor annotation of small genes in reference genomes is also an obstacle of the direct detection from mass spectrometry. Even by combining multiple types of omics data, the false positives can still be high in small bacterial peptides detection (Miravet-Verde et al., 2019). Second, the protein calling tools for metagenomics may require high quality of the assembly results. Especially some of them are optimized for long contigs and scaffolds (Hyatt et al., 2012). Recently, a large-scale study for bacterial peptides from metagenomic samples reported more than 4,000 novel small-protein families were found from human microbiome and less than 5% of the proteins could be mapped to known domains. However, they still used contigs as input data from the nucleotide-level metagenomic assembly, which can lose a large amount of original sequencing data due to the sample complexity and sparsity. Third, for homologous searching and function annotation (Cantalapiedra et al., 2021), there is not a specific tool designed for exploring and mapping to the space of small bacterial peptides.

In order to address the limitations from the nucleotide-level metagenomic assembly and the current shortages of small protein annotation from microbe communities, metaBP (Bacterial Peptides for metagenomic sample) has been developed as a comprehensive and user-friendly toolkit to explore the small protein universe in a more thorough and detailed way. The metaBP applies protein-level assembly from the metagenomic sequencing data to maximize the protein recovery and search from the open reading frames (Steinegger et al., 2019). The metaBP identifies confident small protein sequences and mutations in diverse homologous clusters using the most current protein sequence clustering technique (Steinegger and Söding, 2018). The metaBP also contains a machine learning part, metaBP-ML, to address the sequence-based annotation integrating a natural language-based protein embedding model (Rives et al., 2021) with a million-sized database. Diverse small protein sequences and functions are demonstrated in various sets of samples, which cover mice, human, and environmental microbiome communities. The metaBP provides the capability to explore the small protein landscape both at the microbial community scale and at the base pair resolution.

## 2 Materials and Methods

### 2.1 Toolkit Implementation Overview

**FIGURE 1:**
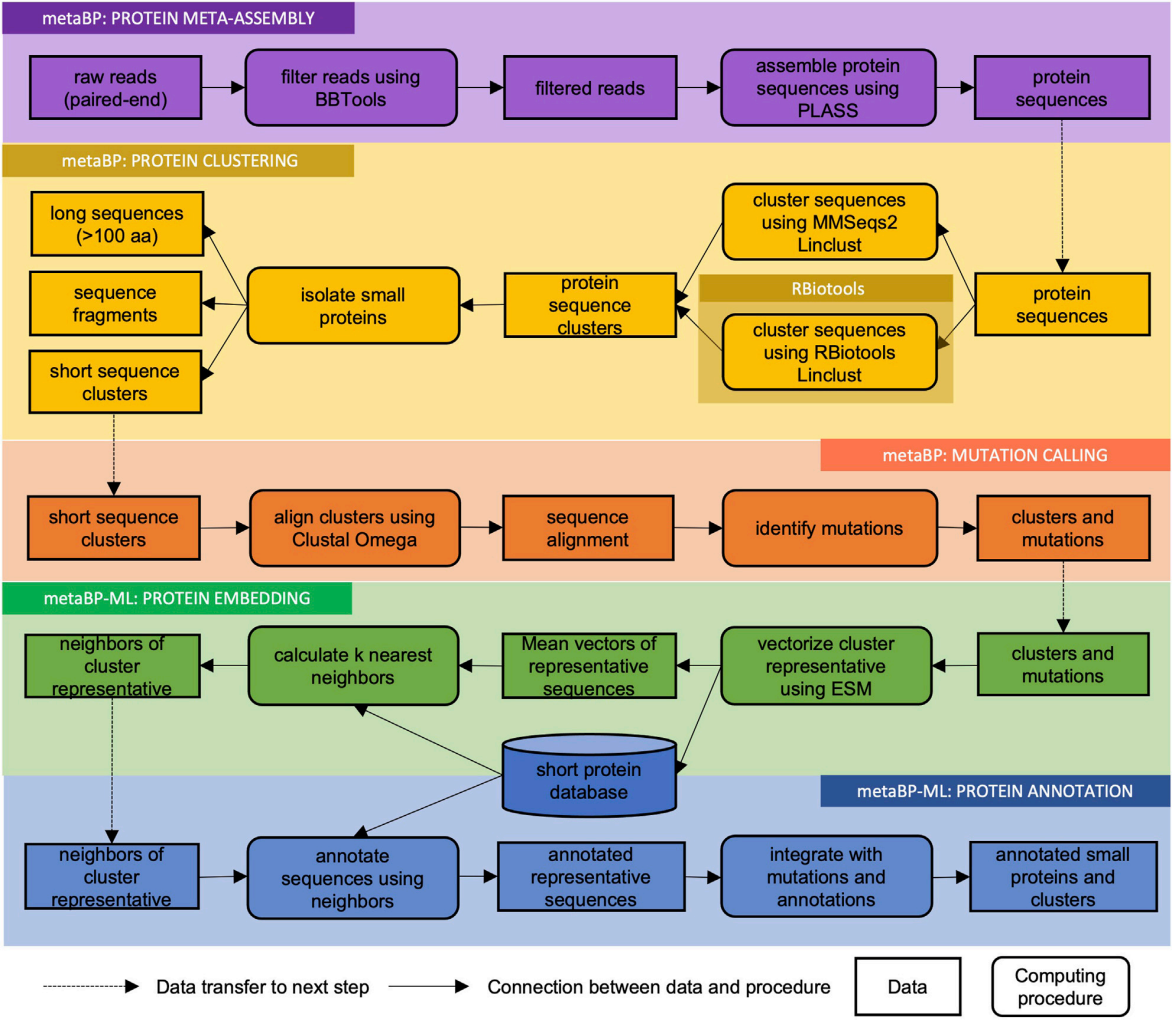
Flowchart of metaBP pipeline. MetaBP’s implementation consists of three major modules (metaBP, metaBP-ML, and RBiotools), and five main procedures (protein meta-assembly, protein clustering, mutation calling, protein embedding, and protein annotation).

The metaBP is an integrated and automated toolkit for identifying and annotating small proteins from the metagenomic sequencing data. MetaBP’s implementation consists of three major modules (metaBP, metaBP-ML, and RBiotools), and five main procedures (Figure 1): protein meta-assembly, protein clustering, mutation calling, protein embedding, and protein annotation. The first three procedures to identify small proteins along with mutations are from our major module, i.e., metaBP; the last two procedures to do protein embedding and annotation are integrated in our machine learning-based module metaBP-ML. The entire toolkit is implemented by both Python and R, and the machine learning module requires pyTorch. The most convenient way to install and use the metaBP, metaBP-ML and RBiotools is to configure their individual Conda environment, which are described in our GitHub repository (see the data availability for our GitHub link).

### 2.2 Input and Output

The input data for metaBP is the raw sequencing reads (paired-end short gun sequencing) in a FASTQ format. The output data consists of mainly three parts of the information, protein clusters with mutations, small protein annotations, and a protein copy number table from annotations, which will be demonstrated in this study. For the purpose of this study, only the small protein analysis is mentioned and emphasized. In fact, the metaBP toolkit can also identify the entire proteome wide space of open reading frames, other than just small proteins.

The raw FASTQ files used in this study are downloaded from NCBI SRA by the sra-toolkit. The sample IDs and general sequencing information are summarized in the supplementary table (Supplementary Table S1), with different read lengths and data volumes. This indicates our metaBP can be generalized to all types of metagenomics from natural environments.

### 2.3 Small Protein Identification and Clustering by MetaBP

#### 2.3.1 Protein-Level Meta-Assembly

Raw sequencing reads in FASTQ files are pre-processed by BBTools (Bushnell et al., 2017). The pre-processing includes quality checking, read length trimming, and adaptor removal. The cleaned reads are used in the protein level assembly by PLASS (Steinegger et al., 2019), which is reported to increase the protein yields by many-folds compared to the nucleotide-level metagenomic assembly. When an example data set from PLASS GitHub (see the data availability) is used to do protein level assembly, 99% identity in the sequence only yields 780 proteins, while 90% and 80% identity yield 1,217 and 1,267 proteins, respectively (Supplementary Table S2). Specifically, 80% of the sequence identity triples the number of non-single clusters. In order to maximize the initial protein throughput and capture the diversity inside average protein clusters, 80% identity in the sequence is recommended using in the metaBP toolkit. This setting can be changed by user’s specific needs in terms of the protein recovery volume.

#### 2.3.2 Protein Clustering and Mutation Calling

The assembled protein sequences are used in the clustering process. Linclust is one of the most recent protein clustering techniques that can approach both the good accuracy and linear time complexity (Steinegger and Söding, 2018). The metaBP has two ways to call Linclust procedure: one is from MMSeqs2 command line, and the other is from our independent implementation in RBiotools. These two different ways to call Linclust provide different flexibility to the user side. The R version of Linclust inside the RBiotools does require additional installation of the R environment, but it is more flexible for user to develop new applications and change parameters from the source code.

The protein clustering by Linclust has two purposes: one is to remove the redundancy from proteins and protein fragments, and the other is to group protein families by homology for mutations. The protein sequences generated from the PLASS example dataset are duplicated to test the effects of truncated sequences. Each protein is truncated up to 50% of the total length from either beginning or end of the sequence, mixed with their intact versions, and then they are clustered by Linclust at different settings. When the default parameter for Linclust is applied, the sequences truncated to 90% of the original length can still be clustered with its full-length version, but sequences truncated to 80% of the length cannot be clustered well. When using the customized setting in Linclust and setting the coverage rate to 50%, most of the truncated protein can still be clustered with the original full-length protein (Supplementary Table S3). For small protein clustering, the default parameters in Linclust are recommended to use the metaBP in order to make the small protein families more specific and sensitive. The user can always change the parameters to accommodate various protein lengths in a cluster. After the protein clusters are generated, the protein sequences are aligned by Clustal Omega (Sievers and Higgins, 2018), and the positions with conservative amino acids or the positions with potential mutations can be observed and reported. By randomly mutating the protein sequences, it is confirmed that Linclust can capture up to 5% of the sequence mutation in the same cluster (Supplementary Table S4). This implies that the final small-protein clusters obtained from the samples can represent a protein family with diverse sequences of at most five amino acid mutations.

The strategy to isolate small proteins from metagenomics data is as follows. First, sequences with longer than 100 amino acids are separated. Second, short sequences clustered with long sequences are removed so that the protein fragments can be minimized in the final output. Third, in this study, only protein clusters with four or more protein members are considered as confident protein families. This means that the same small protein should occur at least four times in a single sample. In addition, only protein clusters with a large size can display a meaningful sequence diversity. On average, after these criteria are applied to the datasets, less than 5% from the metagenomics data are small bacteria peptides, which is consistent with the study from MAGs (metagenome-assembled genomes) or contigs (Sberro et al., 2019).

### 2.4 Machine Learning-Based Annotation by metaBP-ML

#### 2.4.1 Database Construction

The database for small protein sequences (not more than 100 amino acids) is constructed from the sequence files in the FASTA format downloaded from the Uniprot (Swiss-Prot and TrEMBL, November, 2021) (Bateman et al., 2021). In total, 16,565,616 sequences are downloaded for bacteria, 785,496 for archaea, 1,201,161 for virus, and 596,067 for metagenomics. Among these short sequences, 8,486,746 have species or function annotations. The rest of the 10,661,593 proteins without any annotation (“uncharacterized” or “unannotated”) is removed first. Among annotated small proteins, Linclust is used to remove 80% of redundant sequences by clustering, and 3,682,960 proteins are eventually survived to form our final small protein sequence database.

As a transformer-based machine learning model inspired from natural language processing, ESM (Rives et al., 2021) is used to convert the sequences in the database to numerical vectors. In order to process 3 million of small proteins in the database, parallel computing with multiple threads is used to speed up the procedure. The resulted vectors for each small protein are 1,280 numerical values in length, and the principal components are computed in order to visualize the entire small protein database or landscape in a two-dimensional space. To our knowledge, before our study, this small protein landscape hasn’t ever shown nor used in the small protein annotation.

#### 2.4.2 Protein Embedding and Annotation

After the database is constructed with vectorized small protein sequences, small proteins from metagenomic samples must be processed in the same ESM model (Rives et al., 2021). Each of the small protein cluster is vectorized by its representative sequence and then it can be embedded to the entire small protein universe spanned by the database. For downstream protein annotation, user can select one of the two ways in metaBP-ML. The first one is to use an HMM based tool, i.e., eggNOG (Cantalapiedra et al., 2021), which is for general protein annotations as well as for small proteins. The second method is to search for k nearest neighbors (KNN) from our constructed database for each cluster representative. Since this requires calculating all pairs of vector distances, it can be time consuming for a larger k. From our simple test, using a mice gut microbial sample (Morissette et al., 2020), the newly recovered protein annotations drops to less than 10% when pursuing 10 neighbors (Supplementary Table S5). In metaBP-ML, top ten nearest neighbors are recommended for small protein annotations.

The final protein annotation strategy based on the ten nearest neighbors is heuristic. First, rule of thumb is used if there is a most frequent annotation in the neighborhood. Second, if there is no difference between annotation frequencies, the top annotation is always picked. Third, if useful annotation cannot be extracted from the top ten neighbors, the protein will be left as unannotated.

In this study, the enzyme commission (EC) number and the taxonomy information will be provided in the small protein annotation. For simplicity of this research, protein copy numbers are used to quantify the abundance of every annotation so that different samples are compared. The protein copy numbers are added together from different clusters with the same annotation. The protein copy numbers can be normalized by the total number of small protein copies in the data set. The normalized protein copy number [or counts, denotated as c(.)] for a certain annotation A is calculated with the following formula, where s(ℂ) is the size of cluster ℂ . Analogous to transcriptome quantification, the normalized value can be multiplied by 10^6^ to represent the copy numbers per million proteins.

$$c\left(A\right)=\sum_{\mathbb{C}\in A}s\left(\mathbb{C}\right)\times 10^{6}/\sum_{\mathbb{C}}s\left(\mathbb{C}\right).$$

## 3 Results

### 3.1 Small Protein Identification by metaBP in a Wide Range of Samples

The metaBP is applied on various metagenomic data sets, including sixteen mice gut samples (Morissette et al., 2020), one human gut sample (Lee et al., 2017), one human skin sample, one saliva sample, and environmental microbe samples in soil and marine (see Supplementary Table S1 for NCBI Bioproject IDs). The data size varies from 5 million to 45 million of sequencing reads (Table 1; Supplementary Table S1). The sequencing read length varies between 100 and 200 base pairs from each end. On average, about one third of the resulted sequences are short sequences from the protein assembly results. However, only less than 5% of the sequences are in a cluster with at least four sequences, which is consistent with previously reported percentage of small open reading frames in metagenomic samples. Thus, clusters with at least four sequences are treated as reliable small protein families in the sample. Overall, about 5,000–8,000 clusters with their small representative proteins are generated within every million of the total assembled proteins. These clusters and representative sequences are sent to metaBP-ML (and/or eggNOG) for annotation, so that the taxonomy and enzyme commission (EC) information can be obtained and quantified for each sample.

**TABLE 1:** Data samples and statistics in metaBP analyses.

Sample	Biosample	Reads structure	# of reads (m)	# of assembled total proteins	# of small protein clusters with 4 or more members	Time for protein assembly (HH:MM:SS)	Time for metaBP-ML annotation (DD-HH:MM:SS)
1.1	Mice gut	2 × 100 bp	32.7	1847781	16475	00:56:55	01:06:28:23
1.2	Mice gut	2 × 100 bp	29.8	1969398	12725	00:59:14	23:04:32
1.3	Mice gut	2 × 100 bp	25.9	1714448	12916	00:48:12	01:00:06:49
1.4	Mice gut	2 × 100 bp	32.0	1705080	11870	00:56:49	22:07:28
1.5	Mice gut	2 × 100 bp	30.1	1662298	15140	00:49:58	16:48:22
1.6	Mice gut	2 × 100 bp	32.3	1999701	14555	01:03:55	01:15:22:07
1.7	Mice gut	2 × 100 bp	28.3	1525231	12758	00:49:56	01:00:56:54
1.8	Mice gut	2 × 100 bp	37.4	2483994	18411	01:14:25	12:58:13
1.9	Mice gut	2 × 100 bp	33.0	1948886	14380	01:00:10	01:02:03:12
1.10	Mice gut	2 × 100 bp	33.4	2808475	18745	01:23:08	01:08:10:31
1.11	Mice gut	2 × 100 bp	33.7	2167085	16087	01:02:30	01:07:40:41
1.12	Mice gut	2 × 100 bp	35.6	2376492	15779	01:12:33	01:04:21:47
1.13	Mice gut	2 × 100 bp	37.0	2974313	17935	01:39:31	01:07:51:20
1.14	Mice gut	2 × 100 bp	44.5	3626380	19818	01:53:51	12:02:22
1.15	Mice gut	2 × 100 bp	32.5	1965315	16659	01:02:31	01:05:44:15
1.16	Mice gut	2 × 100 bp	31.1	2322776	12481	01:12:24	22:28:17
2	Human gut	2 × 151 bp	37.5	29194727	171238	01:26:04.66	20:15:30:00
3	Human skin	2 × 150 bp	5.1	679115	2874	00:30:24.91	04:54:01
4	Meadowsoil	2 × 200 bp	24.7	14947269	23640	14:41:28.71	01:14:07:31
5	Marine	2 × 150 bp	13.1	4595883	23410	02:12:55.85	01:14:22:30
6	Human saliva	2 × 126 bp	26.8	133743	583	00:36:04.86	01:06:08

**FIGURE 2:**
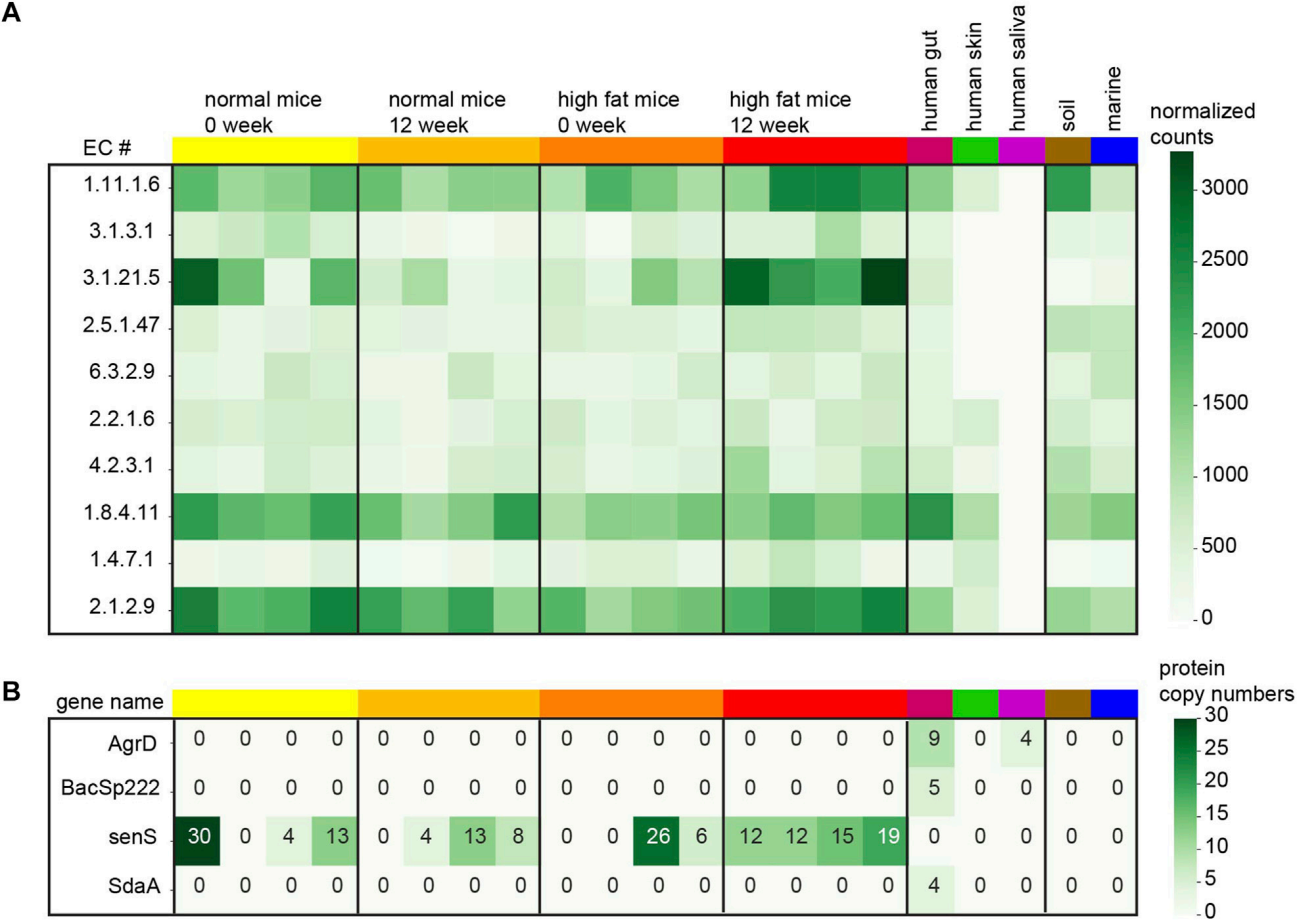
Small protein quantification in each sample. **(A)** Normalized counts (small proteins per million) for top ten EC numbers comparing the high-fat diet mice with the normal mice after 12 weeks. **(B)** Protein copy numbers for 4 known small genes recovered from the samples.

The analysis from mice samples shows interesting enzyme activities. In order to compare EC numbers across samples, only those ECs existing in all the samples are used in this analysis. First, the normalized counts of every EC number from mice samples are tested by ANOVA and the top ten important EC functions enriched in the high-fat diet of 12-week-old mice are presented in the heatmap (Figure 2A). The complete EC quantification table is available in the Supplementary Table S6. These top ten ECs corresponding to the high-fat diet mice show potential enzyme activities from small protein families. For example, the proteins marked with EC 1.11.1.6 belong to the catalase which is important for radical degradation. Catalases and antioxidant enzymes are known to increase in order to benefit the mice with a high-fat diet (Liang et al., 2015; Piao et al., 2017). It is necessary to mention that after the Benjamini–Hochberg *p*-value correction, none of the EC numbers are significant in the high-fat diet mice anymore. So, the EC numbers displayed in the heatmap are simply ranked by its original *p*-value (less than 5%). It is noticeable that the quantification pattern of these EC numbers from the human gut sample is more like the mice gut samples compared with the other samples. Human saliva samples do not have good yield of small proteins compared to the other samples.

In this study, 29 of the known short proteins derived from bacteria are searched from the metaBP output and only four of those are discovered in our samples (Figure 2B). The Uniprot IDs of these small genes are listed in the Supplementary Table S7. The most abundant small genes, senS are discovered in 12 of the 16 mice gut samples, but not in the human gut. The other three genes, AgrD, BacSp222, and SdaA are only recovered from the human gut sample. Indeed these 29 small genes are all from human associated microbes (Sberro et al., 2019) so that they may not be easily observed in the soil and marine samples. While metaBP-ML has discovered four of these 29 genes in our samples, the annotation from eggNOG does not show any of these 29 genes.

### 3.2 Small Protein Annotation by eggNOG and metaBP-ML

Besides the search for the known 29 small genes, sixteen mice gut samples are used to systematically compare the annotation outcomes from eggNOG and metaBP-ML for small protein families. As known that not many small proteins have clear enzyme activities, EC number annotation overall has lower yields compared with the taxonomy (organism group) annotation, no matter by eggNOG or metaBP-ML.

**FIGURE 3:**
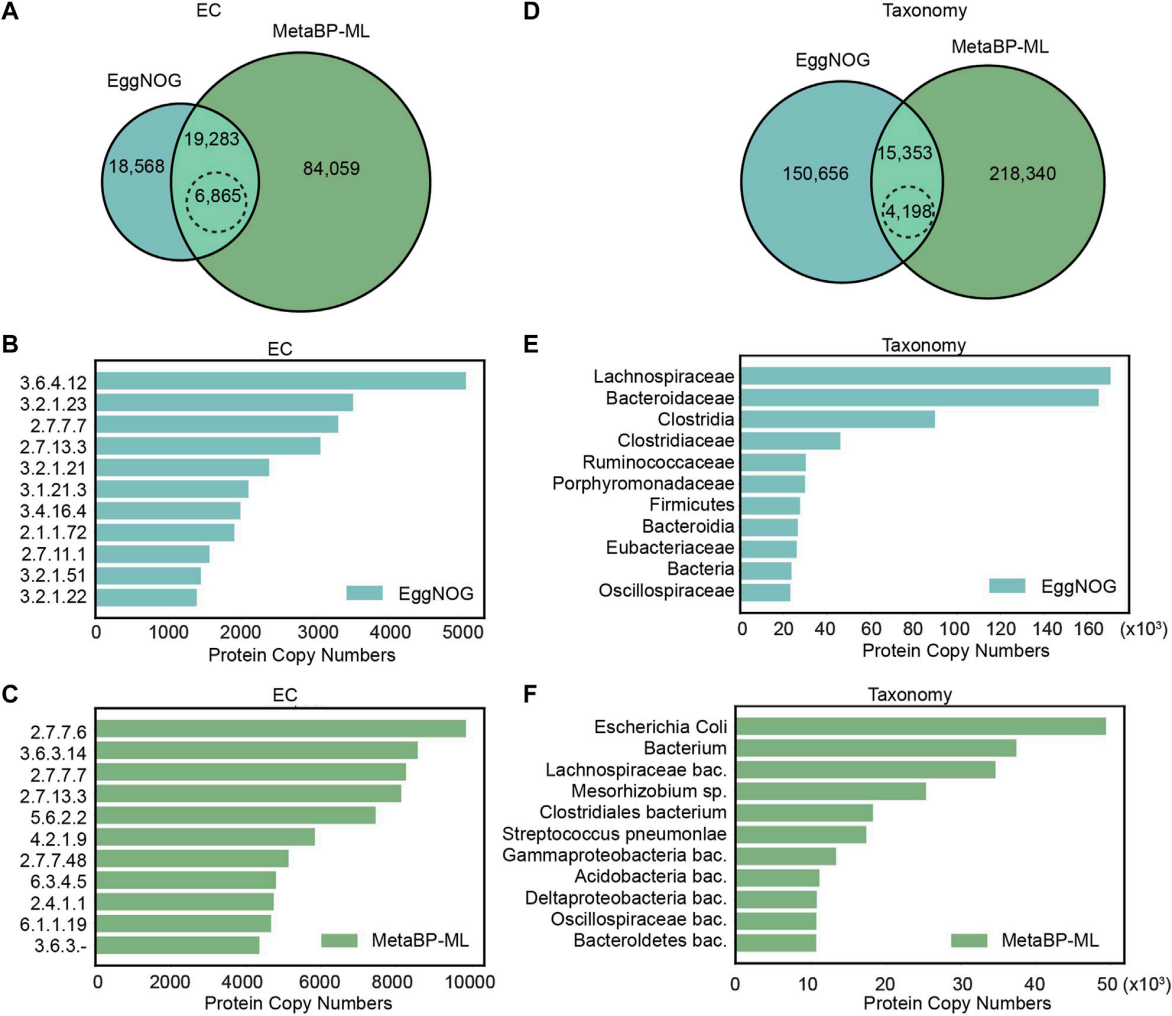
Comparison of small protein annotations from eggNOG and metaBP-ML. **(A)** Number of proteins can be annotated with EC numbers by eggNOG and metaBP-ML. A total of 6,865 proteins in dashed edge circle are annotated with exactly the same EC numbers. **(B)** Top 11 EC numbers predicted from eggNOG **(C)** Top 11 EC numbers predicted from metaBP-ML. **(D)** Number of proteins can be annotated with taxonomy terms by eggNOG and metaBP-ML. A total of 4,198 proteins in the dashed edge circle are annotated with exactly the same family names. **(E)** Top 11 taxonomy terms predicted from eggNOG. **(F)** Top 11 taxonomy terms predicted by metaBP-ML.

For the EC number annotation, metaBP-ML can annotate almost five times more proteins than eggNOG (Figure 3A). Both methods can annotate the same set of 19,283 proteins, but 6,865 proteins have the consensus EC annotation. Among the top 11, the most abundant EC numbers in eggNOG and metaBP-ML (Figures 3B,C), EC2.7.7.7 (DNA-directed DNA polymerase) and EC2.7.13.3 (histidine kinase), occur in both methods. However, it is hard to confirm if the small proteins can have these enzyme activities or not, since the functions are assigned only by the similarity computation.

For taxonomy annotation, metaBP-ML can annotate almost twice of the proteins than eggNOG (Figure 3D). In order to compare the predicted taxonomy labels directly, taxonomy IDs from both the methods are normalized to family IDs. This means among the same set of 15,353 proteins that gain the taxonomy annotation from both the methods, only 4,198 proteins have exactly the same family name from both the methods. The consensus rate is between 1/3 to 1/4 between two approaches. Top 11 abundant taxa from eggNOG are family names, order names or phylum names (extracted from the narrowest annotation from eggNOG results), while top 11 taxa from metaBP-ML, which can be as detailed as species level annotation (Figures 3E,F). From the top taxa lists obtained in both the methods, Lachnospiraceae, Oscillospiraceae, and Clostridia are the consensus. Overall, our metaBP-ML can provide more annotations with more details mainly because of a very specific small protein database constructed.

### 3.3 Small Protein Landscape by metaBP-ML

**FIGURE 4:**
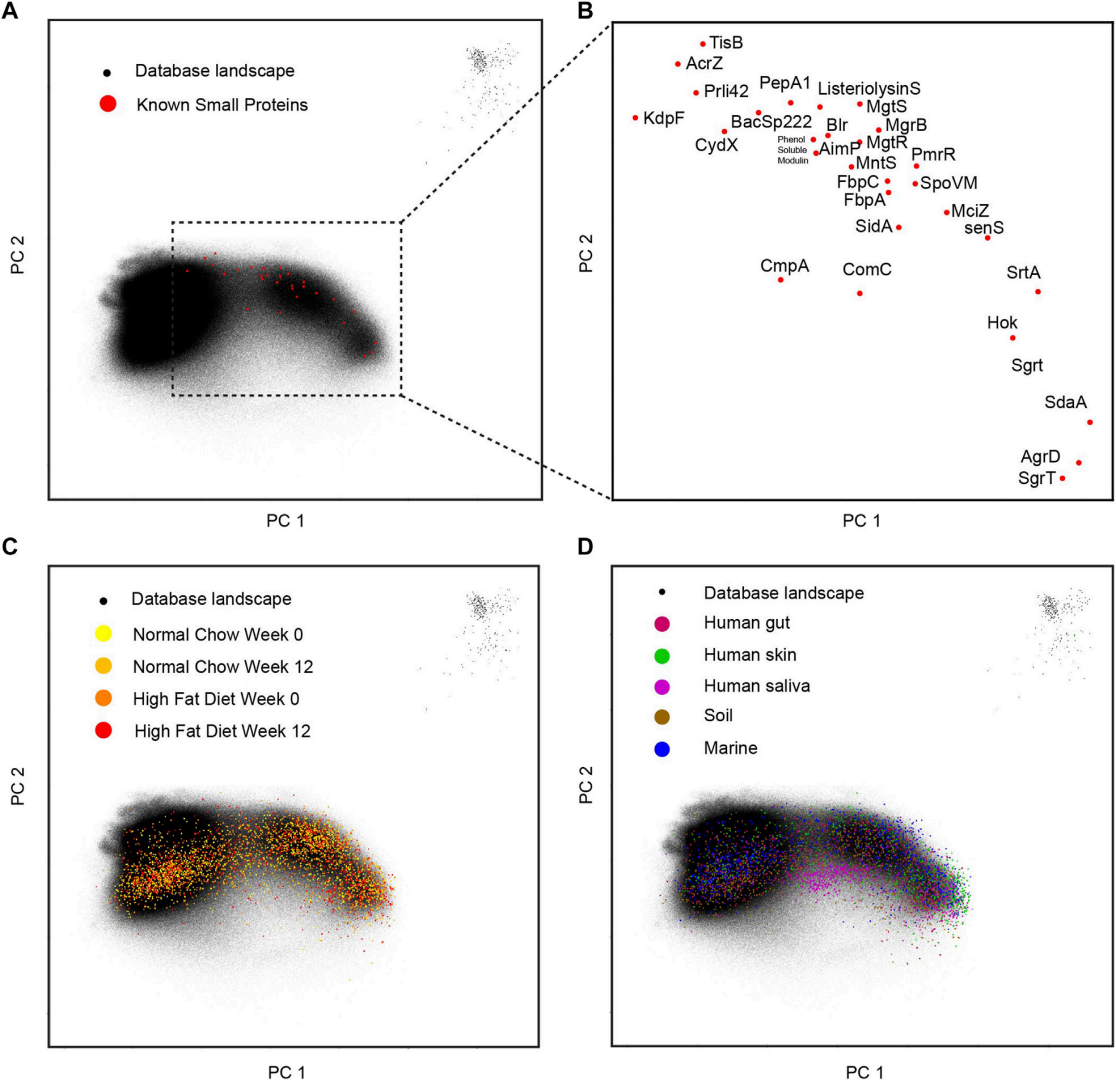
Landscape for small proteins. **(A)** The database with 29-known small proteins overlaid. **(B)** The zoomed-in display for 29-known small proteins in the two-dimensional space. **(C)** The different mice gut samples overlay with the database landscape. **(D)** The human and environmental samples overlay with the database landscape.

As mentioned above, the entire small protein database composed of 3 million of short sequences are transformed into a 1,280-dimension vector space. In order to visualize the landscape within two dimensions, principal components analysis is performed, and the first two principal dimensions are shown in a dot plot (Figure 4A). The collected 29-known small genes are overlaid on this landscape and their relative locations and gene names are in a zoomed-in plot (Figure 4B). Surprisingly, within the first two principal components, the small protein landscape clearly shows three clusters: left, right, and some outliers on the top right corner. It is hard to tell if this pattern of distribution reflects the true biology or some artifacts in the data collection, which requires future investigation. The known 29 small genes are mainly located on the right side of the landscape. When the mice samples are overlaid to this landscape (Figure 4C), there is no observable sample effects. When more samples are overlaid onto this landscape (Figure 4D), we can observe that the soil sample and skin sample are more on the right side while the human saliva sample is more located under the conjunction of the two parts. This entire landscape built from small proteins makes it possible to visualize the sample specific patterns from a natural microbial community.

### 3.4 Sequence Diversity in Small Protein Clusters

**FIGURE 5:**
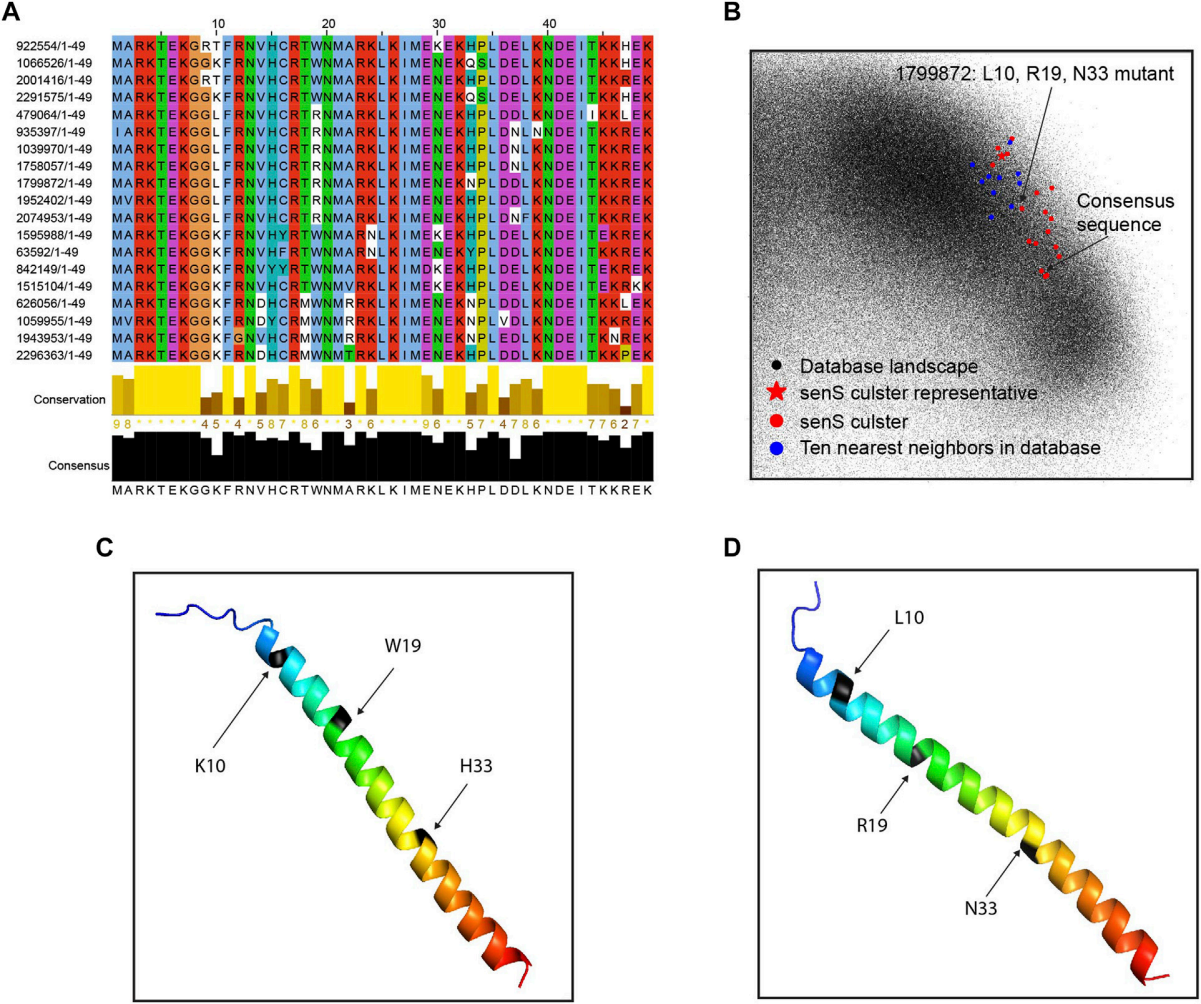
Sequence diversity of senS gene. **(A)** Sequence alignment and conservation of the senS proteins. **(B)** The senS cluster and ten neighbors overlay onto the database landscape. **(C)** The predicted structure for the consensus sequence of senS. **(D)** The predicted structure for a mutant of the consensus.

To explore several interesting clusters identified in the mice gut samples, we pull out the protein cluster sequences from metaBP results and conduct further analyses. The clusters shown in this section are from the 12-week-old mice with a high-fat diet. One of the known small genes, senS, is widely discovered in the mice gut samples, and its sequence diversity is shown after the sequence alignment (Figure 5A). The senS protein sequences, including the consensus sequence and one of the mutants, are overlaid with all small proteins (Figure 5B). This cluster is located on the right side of the landscape (Supplementary Figure S1). By using Alphafold2 (Jumper et al., 2021), the consensus sequence of senS is predicted as an alpha helix structure (Figure 5C). Having three amino acids mutations, the structure for the mutant protein still shows a clear helix, but with a slightly bending effect (Figure 5D).

Another interesting cluster is from catalase EC1.11.1.6. The alignment of the sequences shows very few possible mutations are detected in the high-fat diet mice (Supplementary Figure S2). The structures predicted by alphafold2, as well as the display of the protein landscape, show that the two amino acids substitution with longer side chains (R vs. G, N vs. H) help to make the loop region a little bit more structured, but not too much overall change. The structures show an alpha helix and beta sheet motif for this protein cluster.

## 4 Discussion

The metaBP adopts protein level assembly by PLASS, and therefore it is not constraint by the requirement of long contigs or high-quality MAGs from the nucleotide level assembly. As we know, low-abundant rare species may overall constitute a large amount of the sequencing reads in the complex metagenomic samples but may not yield long contigs. When the sequencing depth is low, more than half of the data could be wasted as unassembled sequencing reads. But for small proteins this fragmented sequencing data should already provide sufficient information for both the sequence and function. The metaBP together with metaBP-ML provide users with a complete toolkit to explore small proteins in natural metagenomic samples. For potential extension, the metaBP-ML does allow users to build their application specific models for protein annotation. In addition to metaBP-ML, we still provide eggNOG in the package to annotate proteins alternatively. In terms of the running time, eggNOG is more efficient with their pre-built reference database. The metaBP-ML is relatively taking more time when annotating proteins through vectorization and nearest neighbors. But due to our constructed small protein database, metaBP-ML can be very specific to identify and annotate small proteins. With the integration of both the tools, the metaBP can be used in various kinds of metagenomic data and annotate arbitrary protein classes.

However, there are still concerns and limitations from the current version of metaBP. Clusters with singletons at this moment are not used for the downstream analysis in the current metaBP. We assume that only re-occurred sequences within the same cluster can indicate the reliability of small proteins and their mutations. Generally, high quality metagenomic data should be sufficient in the sequence depth. However, in many unexpected cases, metagenomic dataset can be sparse, and the clusters with lower number of protein members can also be informative for small proteins. Computationally, there has not been a perfect strategy to balance the false positives and false negatives without knowing the ground truth in the real data sets. But with the metaBP, we can at least provide a short list for the experimental detection through mass spectrometry and biochemical analysis.

The metaBP quantify the annotated features using the normalized protein copy numbers. Due to the protein level assembly, the protein copy numbers are the most straightforward quantification obtained from the data set. Although metaBP can recover more annotations than eggNOG, the quantification may not be sufficient to statistically recover significant features when comparing the samples. One future direction is to improve the resolution of the quantification using the original sequencing reads. The metaBP also displays the protein diversity by homologous protein clustering, but the current metaBP cannot quantify the confidence level of each amino acid mutation. So, the current metaBP is only for the discovery of the potential sequence diversity in a protein family, not for the strict quantification of mutation occurrence.

## 5 Conclusion

This study proposes a new and comprehensive toolkit, metaBP (and metaBP-ML), to discover and annotate the community specific bacterial (microbe derived) peptides from the metagenomic samples. It is built upon a new idea of direct protein level assembly and one of the current protein clustering tools, as well as machine learning based approaches. The exploration of the small protein landscape and the analyses of peptides annotation demonstrate the efficacy of this work and the value of machine learning.

## Data Availability

Publicly available metagenomic datasets were analyzed in this study. These data can be downloaded from NCBI SRA repository by the information provided in Supplementary Table S1. The small example data set for testing parameters are from PLASS GitHub (https://github.com/soedinglab/plass/tree/master/examples). The pipeline and tools are available through github for metaBP (https://github.com/yao-laboratory/metaBP), metaBP-ML (https://github.com/yao-laboratory/metaBP_ML) together with an integrated version of RBiotools. In metaBP-ML, ESM and its model are used. The source codes and models can be found from ESM GitHub: https://github.com/facebookresearch/esm. The pre-trained model for general purpose “esm1b_t33_650M_UR50S” is used for this proposed embedding work.
