# Supplementary figures and images for: Profiling a Community-Specific Function Landscape for Bacterial Peptides Through Protein-Level Meta-Assembly and Machine Learning
Source: Front Genet. 2022 Jul 22;13:935351. doi: 10.3389/fgene.2022.935351 (PMC9354662; doi:10.3389/fgene.2022.935351)

Supplementary Figure 2

A

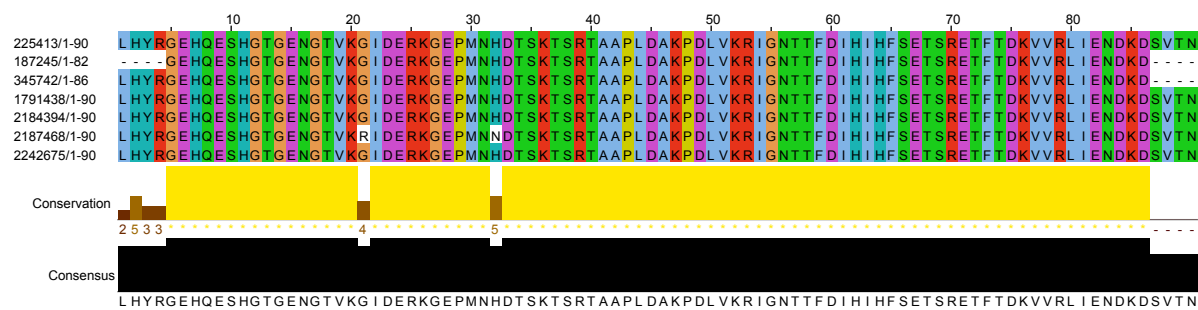

B

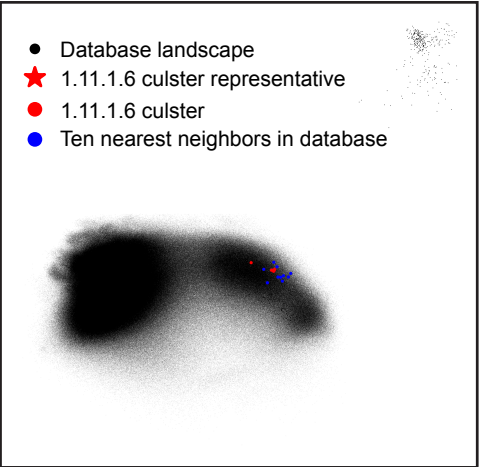

C

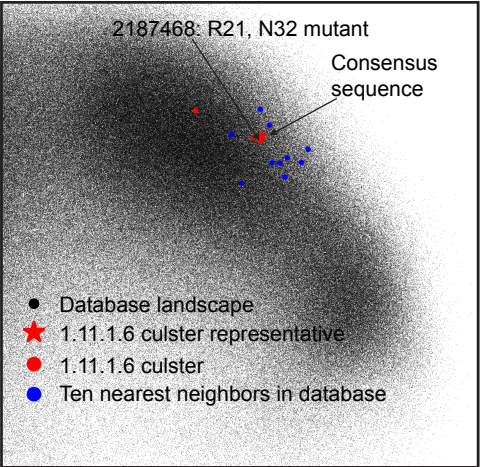

D

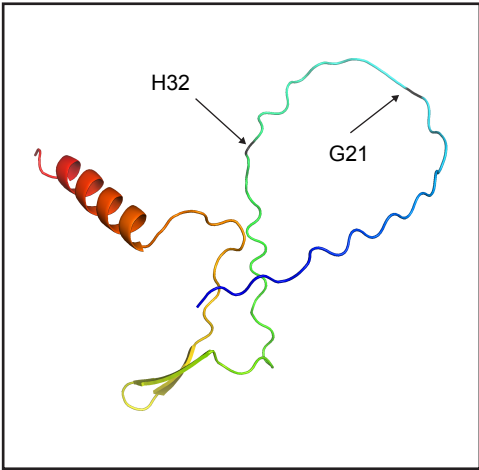

E

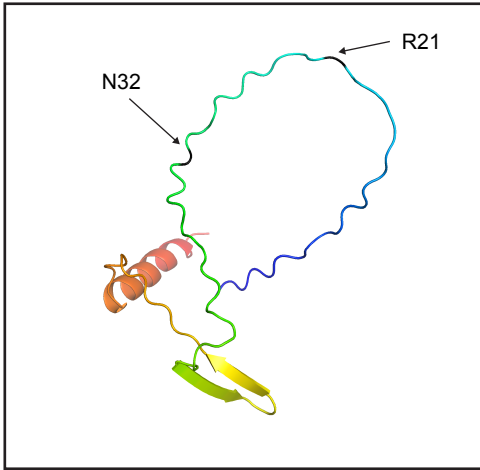

Supplement: Supplementary file 3 [file Image2.PDF]

Supplementary Figure 1

A

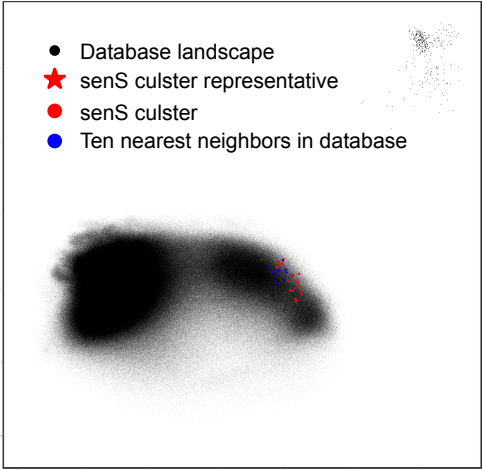

B

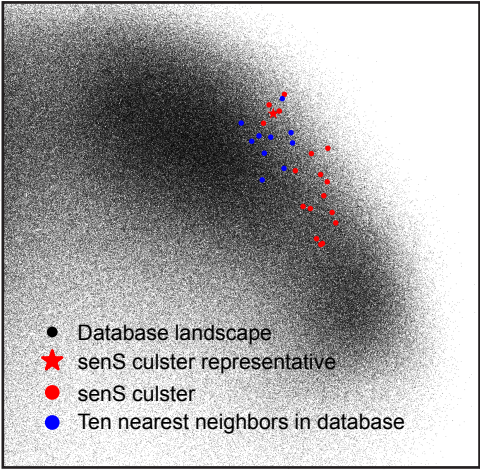

Supplement: Supplementary file 10 [file Image1.PDF]
