# Supplementary material for: Profiling a Community-Specific Function Landscape for Bacterial Peptides Through Protein-Level Meta-Assembly and Machine Learning
Source: Front Genet. 2022 Jul 22;13:935351. doi: 10.3389/fgene.2022.935351 (PMC9354662; doi:10.3389/fgene.2022.935351)
Supplement: Supplementary file 5 [file DataSheet1.PDF]

# Profiling community-specific function landscape for bacterial peptides through protein level meta-assembly and machine learning

## *Supplementary Material*

## 1 Supplementary Data and Information

### 1.1 MetaBP Usability

MetaBP is the major pipeline to perform the protein level assembly and protein clustering. EggNOG can be used to annotate the resulted small proteins if user desires.

MetaBP (version as of 06/2022) can be downloaded from GitHub: [https://github.com/yao-laboratory/metaBP](https://github.com/yao-laboratory/metaBP)

(1) Installation:

MetaBP can be installed by running "install.sh" through a set of conda installation commands. RBiotools is not mandatory to install. If the user desires to use cluster functions and/or other features in RBiotools, please run the "install_rbiotools.sh" to set up the R environment.

(2) Major commands:

MetaBP can be used with two kinds of input files. You can either start with raw sequencing read files (paired-end .fastq files) or protein sequences that are already assembled in a .fasta file.

```
python mutation_pipeline.py call_mutations -i1 R_1.fastq -i2 R_2.fastq -o
metabp_output_folder -clust 0
```

```
python mutation_pipeline.py call_mutations -s all_proteins.fasta -o metabp_
output_folder -clust 0
```

MetaBP command options:

- **-i1 -i2** raw sequencing files (.fastq.gz) for paired-end reads
- **-s** the assembled protein file in .fasta format if the user already run the assembly. In this case PLASS assembly is to be bypassed. This is an alternative input to the –**i1** and –**i2** arguments.
- **-o** the path for the output directory
- **-clust** 0 when RBiotools is not used

(3) Methods explanation and output files from above commands:

These steps are done internally in metaBP and they are explained in the following paragraphs.

First, metaBP takes the raw sequencing data into the preprocessing step to do the quality checking, filtering and adapter removal. The resulting processed reads are passed into PLASS for protein-level assembly. The assembled protein sequences from the PLASS tool are saved in **assembly.fas.**

Second, the assembled sequences are clustered using Linclust. If preassembled sequences are provided, this is the first step in the metaBP. The user has a choice between two linear clustering tools: RBiotools Linclust, where the linear clustering algorithm is implemented in R, or the original Linclust tool from the MMSeqs2 package. The output from both versions of Linclust is reformatted in an additional step to make sure the format is consistent. The fully formatted clusters can be found in the output directory as **clusters_all_seqs.fasta**.

Third, the proteins clusters are sorted through to separate the short proteins from the rest. Proteins with more than 100 amino acids in them are removed and written to a separate file in the output directory named **long_proteins.fasta**. If shorter proteins are in clusters with long proteins, they are considered fragments and written to the **fragments.fasta** file in the output directory. The clusters with short proteins are then rewritten to a new file, **clusters_short_prots.fasta**, and the short sequences (without clusters information) are written to the file **short_prots.fasta** to facilitate the downstream analysis.

Finally, the short clusters are run through the metaBP mutation identification algorithm. Each cluster is aligned using ClustalOmega. Then, each protein in the cluster is compared to the representative sequence to scan for mutations. The mutation positions and amino acids are specified in the headers of the fasta file. Need to mention that our current version is only calling mutations at the relatively conserved protein regions. The mutation information is written to the output directory as **mutations.txt**.

(4) EggNOG annotation for small proteins:

```
emapper.py -i metabp_output_folder/short_prots.fasta -o eggnog_results
```

### 1.2 MetaBP-ML Usability

The motivation of having metaBP-ML separately is due to its own working environment. MetaBP (version as of 06/2022) can be downloaded from GitHub: https://github.com/yao-laboratory/metaBP_ML

(1) Installation:

To setup the conda environment needed to compute the mean vectors, please run:

```
conda env create -f environment.yml
```

In metaBP-ML, ESM and its model are used. The original codes and models can be found from their GitHub: https://github.com/facebookresearch/esm. The pre-trained model for general purpose "esm1b_t33_650M_UR50S" is used for our embedding work.

(2) Major commands:

First, mean vectors must be created for the protein sequences that need annotating using a command such as the following:

```
python compute_bulk_embeddings.py input_protein_file.fasta \
            --output_file all_vectors.txt --batch_size 100
```

The resulted human readable text file has the following format:

```
ProteinID1, [embedded vector 1]
ProteinID2, [embedded vector 2]
```

We recommend the user to submit a job to run this python script since the running time can be long. The example job file can be found as "compute_embeddings.sh".

Second, the resulted text file for protein embedding can be converted to pickle files in order to save space and I/O time in the following steps.

```
python pickle_db.py all_vectors.txt --output_file all_vectors.pkl
```

The target protein sequences can be calculated for embedding vectors using the same script "compute_bulk_embeddings.py". Once these mean vectors have been calculated, the process to assign annotation information can be started using the following command:

```
python metabp_annotations.py get_annotations -i target_protein_vector.txt \
      -o output_directory -db all_vectors.pkl
```

(3) Methods explanation and output files from above commands:

When the above command is run, a k-nearest-neighbors strategy is used to find the k nearest neighbors (k=10) for each target sequence from our database. These protein sequence ids for the neighbors are then written to a file in the output directory provided named **knn_output.csv**. Each row in this file has the id of the protein sequence to be annotated, as well as the ids of the 10 neighbors.

Once the k-nearest-neighbors are found, metaBP pulls the information for each neighbor from the database. The most frequent value for both taxonomic id and EC number in the neighbors is chosen as the best-fit to annotate the protein sequence with. If the neighbors all have differing information, the information of the nearest neighbor is chosen. The taxonomic id and EC number are assigned independently and are written to the **annotated_sequences.csv** file which contains the sequence id, the taxonomic id, the species and the EC number for each target protein.

# 2 Supplementary Figures and Tables

## 2.1 Supplementary Figures

**Supplementary Figure 1.** The senS proteins overlaying onto the database landscape. (A) The embedded positions of the senS cluster and ten nearest neighbors in the database landscape. (B) The zoomed-in plot of (A) , with the consensus and mutant proteins pointed by arrows.

**Supplementary Figure 2**. Sequence diversity in the catalase EC1.11.1.6. (A) Sequence alignment and conservation of the seven catalase proteins in a mice sample. (B) The catalase cluster and ten neighbors overlaid onto the database landscape. (C) The zoomed-in plot of (B), with the consensus and mutant proteins pointed by arrows. (D) The predicted structure for consensus sequence. (D) The predicted structure for a mutant (R21, N32) of the consensus.

## 2.2 Supplementary Tables

**Supplementary Table 2.** Data samples used in this study and statistics from metaBP pipeline.

**Supplementary Table 2.** Minimum sequence identity in PLASS.

**Supplementary Table 3.** Test on Linclust for truncated proteins with default and modified parameters.

**Supplementary Table 4.** Test on Linclust for retaining mutated proteins.

**Supplementary Table 5.** KNN and recovered EC numbers.

**Supplementary Table 6.** EC normalized counts for mice samples and comparison between normal and high fat diet.

**Supplementary Table 7.** Known small proteins and their ID mapping.
